# Machine learning approach to classifying declines of physical function and muscle strength associated with cognitive function in older women: gait characteristics based on three speeds

**DOI:** 10.3389/fpubh.2024.1376736

**Published:** 2024-06-12

**Authors:** Bohyun Kim, Changhong Youm, Hwayoung Park, Hyejin Choi, Sungtae Shin

**Affiliations:** ^1^Department of Health Sciences, The Graduate School of Dong-A University, Busan, Republic of Korea; ^2^Biomechanics Laboratory, Dong-A University, Busan, Republic of Korea; ^3^Department of Mechanical Engineering, College of Engineering, Dong-A University, Busan, Republic of Korea

**Keywords:** dementia, frailty, sarcopenia, machine learning, gait variability

## Abstract

**Background:**

The aging process is associated with a cognitive and physical declines that affects neuromotor control, memory, executive functions, and motor abilities. Previous studies have made efforts to find biomarkers, utilizing complex factors such as gait as indicators of cognitive and physical health in older adults. However, while gait involves various complex factors, such as attention and the integration of sensory input, cognitive-related motor planning and execution, and the musculoskeletal system, research on biomarkers that simultaneously considers multiple factors is scarce. This study aimed to extract gait features through stepwise regression, based on three speeds, and evaluate the accuracy of machine-learning (ML) models based on the selected features to solve classification problems caused by declines in cognitive function (Cog) and physical function (PF), and in Cog and muscle strength (MS).

**Methods:**

Cognitive assessments, five times sit-to-stand, and handgrip strength were performed to evaluate the Cog, PF, and MS of 198 women aged 65 years or older. For gait assessment, all participants walked along a 19-meter straight path at three speeds [preferred walking speed (PWS), slower walking speed (SWS), and faster walking speed (FWS)]. The extracted gait features based on the three speeds were selected using stepwise regression.

**Results:**

The ML model accuracies were revealed as follows: 91.2% for the random forest model when using all gait features and 91.9% when using the three features (walking speed and coefficient of variation of the left double support phase at FWS and the right double support phase at SWS) selected for the Cog+PF+ and Cog–PF– classification. In addition, support vector machine showed a Cog+MS+ and Cog–MS– classification problem with 93.6% accuracy when using all gait features and two selected features (left step time at PWS and gait asymmetry at SWS).

**Conclusion:**

Our study provides insights into the gait characteristics of older women with decreased Cog, PF, and MS, based on the three walking speeds and ML analysis using selected gait features, and may help improve objective classification and evaluation according to declines in Cog, PF, and MS among older women.

## 1 Introduction

Cognitive and physical function declines with natural aging (1), such as the degeneration of the neuromotor control system of the central nervous system (2). Age-related brain pathological disorders cause declines in memory, executive function, visuospatial function, and processing and the prevalence of cognitive impairment increases with age (1, 3). Furthermore, age-related changes in muscle structure, as well as and volume loss in brain structures associated with cognition and movement, contribute to a declines in muscle strength and function (2, 4, 5). Previous studies associated with cognitive function decline in older adults have reported decreased gait speed (6) and handgrip strength (HGS) (7), and increased time taken in five times sit-to-stand (FSTS) tests (8), as parameters of the physical function in older adults as well as in those with sarcopenia (9, 10). This result may be due to decreased movement ability because of the decreased motor planning and executive function with age (11). Impairments of the motor system, such as gait abnormality and low physical function and muscle strength levels, precede the onset of cognitive decline with age (6, 8) or occur during the early stages of dementia (12). Despite these considerations, there remains a significant gap in the studies that simultaneously evaluate the interplay of cognitive function (Cog), muscle strength (MS), and physical function (PF) based on gait analysis.

Gait is a controlled task that requires a high attention level and integration of sensory input, cognitive-related motor planning and execution, and the musculoskeletal system (11). Gait speed predicts declines in Cog, PF, and MS associated with aging (6, 13, 14); however, it is unlikely to be sensitive enough to indicate subtle changes in Cog, PF, and MS (15, 16). Previous studies on gait characteristics in older adults with Cog, PF, and MS declines reported that these characteristics were significantly associated with spatiotemporal variables, phase variables, and gait variability (GV) (12, 17–19). However, these studies measured speed on a short walkway of 4 −10 m (12, 17–19), obtaining uncontrolled walking speed with quantitative values (18, 19). In addition, most previous studies utilized averaged data from repeated trials to gain multiple steps, a practice that may not consistently replicate the natural gait patterns of individuals (20). Therefore, analyzing gait using continuous steps at slow, fast, and self-preferred speeds may be beneficial when evaluating the declines in Cog, PF, and MS among older adults.

Moreover, recent studies have used artificial-intelligence-based machine learning (ML) to improve Cog decline detection and classification using gait characteristics in older adults (1, 21, 22). These studies have reported a classification accuracy of approximately 60–96% when support vector machines (SVM) (21, 22), random forests (RF), and artificial neural networks (1) were used for training. However, these ML studies had limitations similar to those mentioned above and were vulnerable to data overfitting risks owing to their high correlation with multiple variables (1, 23). Furthermore, recent studies recommend gait analysis using continuous steps and wearable sensors (1, 16), as well as analysis methods that extract optimized gait characteristics to overcome these limitations (24). While a previous study has demonstrated the capability of wearable sensors to identify Cog decline and physical frailty (25), it did not investigate whether wearable sensors can distinguish between groups with specific impairments, especially those with simultaneous declines in Cog and PF as well as those with declines in both Cog and MS. Thus, research on objective evaluation using optimized gait feature extraction methods to predict declines in PF and MS simultaneously during the early stages of Cog decline is needed.

Therefore, this study aimed to (1) use stepwise regression to extract gait features based on three walking speeds and (2) evaluate the accuracy of ML models based on the selected features to solve classification problems caused by declines in PF and MS with Cog decline. We hypothesize that analyzing the gait features in the three walking speeds will demonstrate objective classification accuracy based on the differences in gait characteristics between groups with declines in Cog and PF and healthy groups, as well as between groups with declines in Cog and MS and healthy groups.

## 2 Methods

### 2.1 Participants

**Figure 1:**
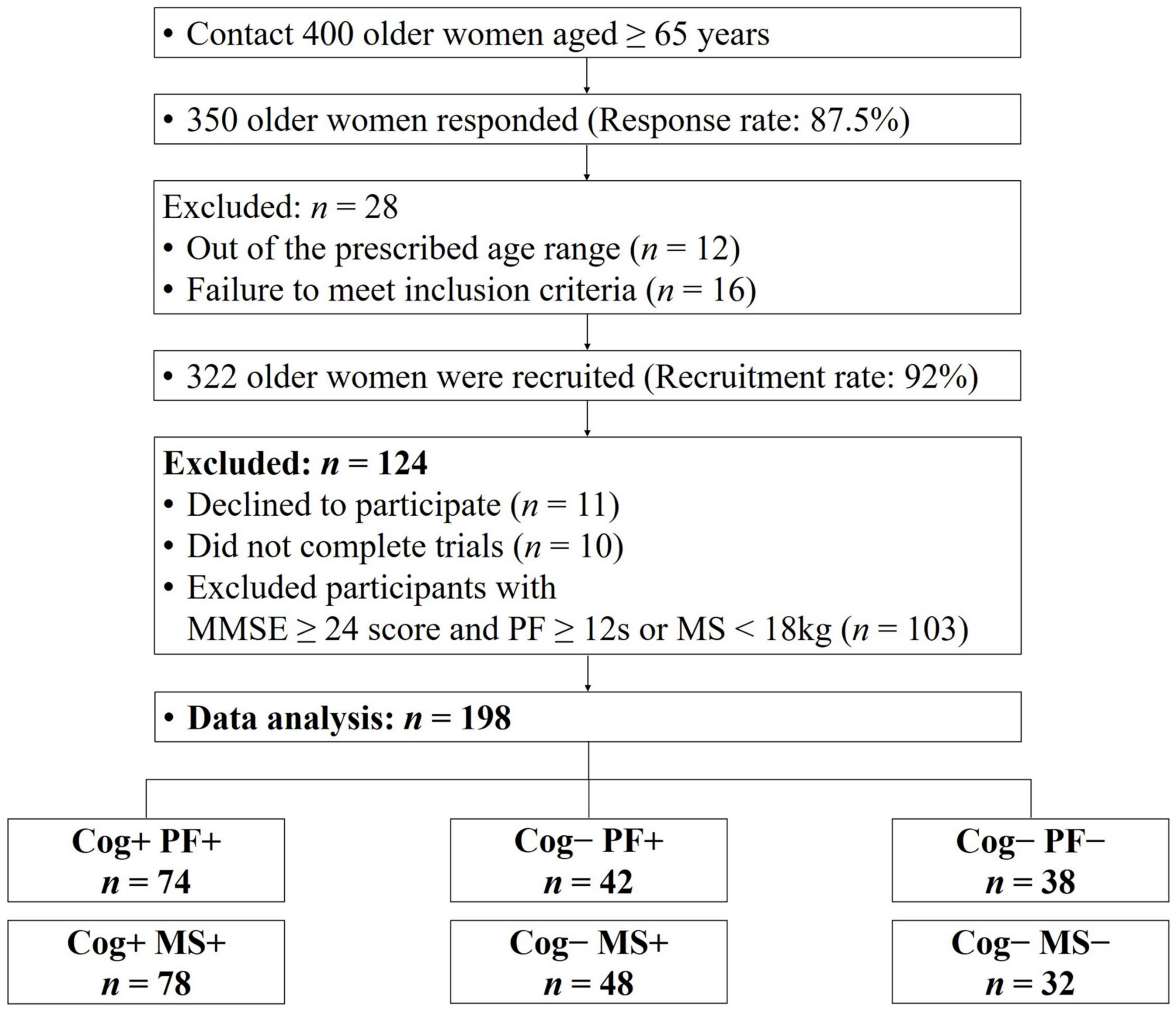
Consort flow diagram. MMSE, minimental state examination; Cog, cognitive function; PF, physical function; MS, muscle strength.

Participants in this study were 198 women aged 65 years or older. The number of participants was calculated using the G-power test program, with the following criteria: effect size of 0.25, significance level of 0.05, power of 0.8, and number of groups of 3. A total of 159 participants was required. Figure 1 presents a flowchart containing the details of the study participants, and their demographic and physical characteristics are shown in Tables 1, 2. This study’s participant inclusion criterion was the ability to walk and move independently. The exclusion criteria included participants with a history of cardiovascular, musculoskeletal, vestibular, or other neurological disorders in the last 6 months and those requiring assistive devices for movement.

**Table 1:** Demographic and physical characteristics of Cog and PF groups.

Characteristics	Cog + PF+ (*n* = 74)	Cog − PF+ (*n* = 42)	Cog − PF− (*n* = 38)	Cog + PF+ *Vs* Cog − PF+	Cog + PF+ *Vs* Cog − PF−	Cog − PF+ *Vs* Cog − PF−
				*p* value		
Age (years)	71.99 ± 5.18	74.71 ± 5.30	77.82 ± 5.31	0.024	**<0.001**	0.027
Height (cm)	152.47 ± 5.07	152.57 ± 6.33	150.74 ± 6.70	1.000	0.418	0.491
Body weight (kg)	58.66 ± 7.08	57.54 ± 8.21	57.63 ± 9.91	1.000	1.000	1.000
BMI (kg/m^2^)	25.25 ± 2.93	24.65 ± 2.64	25.28 ± 3.58	0.931	1.000	1.000
Skeletal muscle mass (kg)	20.09 ± 2.54	20.45 ± 3.17	19.08 ± 2.84	1.000	0.213	0.090
Body fat mass (kg)	21.11 ± 4.96	19.52 ± 4.78	21.68 ± 6.52	0.381	1.000	0.219
Percent body fat (%)	35.40 ± 5.11	33.37 ± 5.40	36.70 ± 5.77	0.155	0.674	0.018
MMSE (scores)	27.16 ± 1.78	21.17 ± 2.23	19.89 ± 3.00	**<0.001**	**<0.001**	0.039
Five sit-to-stand (s)	8.69 ± 1.90	9.54 ± 1.76	16.16 ± 4.28	1.000	**<0.001**	**<0.001**
Education (years)	12.80 ± 3.45	10.12 ± 1.99	8.82 ± 2.30	0.417	**<0.001**	**<0.001**
IPAQ-SF (MET min/week)	2874.02 ± 2349.09	2972.85 ± 2576.81	1566.53 ± 1734.69	1.000	0.014	0.020

All data represent the mean ± standard deviation; Cog, cognitive function; PF, physical function; BMI, body mass index; MMSE, minimental state examination; IPAQ-SF, international physical activity questionnaire-short form; MET, metabolic equivalents; Boldface indicates significant differences, *p* < 0.0167.

**Table 2:** Demographic and physical characteristics of Cog and MS groups.

Characteristics	Cog + MS+ (*n* = 78)	Cog − MS+ (*n* = 48)	Cog − MS− (*n* = 32)	Cog + MS+ *Vs* Cog − MS+	Cog + MS+ *Vs* Cog − MS−	Cog − MS+ *Vs* Cog − MS−
				*p* value		
Age (years)	72.54 ± 4.84	74.38 ± 4.65	78.91 ± 5.61	0.135	**<0.001**	**<0.001**
Height (cm)	153.87 ± 4.58	153.79 ± 5.50	148.56 ± 6.78	1.000	**<0.001**	**<0.001**
Body weight (kg)	59.66 ± 7.63	59.83 ± 8.44	54.22 ± 8.89	1.000	**0.005**	**0.009**
BMI (kg/m^2^)	25.22 ± 3.25	25.23 ± 2.76	24.53 ± 3.59	1.000	0.906	1.000
Skeletal muscle mass (kg)	20.72 ± 2.45	20.98 ± 2.94	18.03 ± 2.39	1.000	**<0.001**	**<0.001**
Body fat mass (kg)	21.32 ± 5.47	20.94 ± 5.47	19.97 ± 6.17	1.000	0.761	1.000
Percent body fat (%)	34.89 ± 5.88	34.28 ± 5.50	35.96 ± 6.15	1.000	1.000	0.625
MMSE (scores)	27.13 ± 1.78	21.35 ± 1.85	19.38 ± 3.28	**<0.001**	**<0.001**	**<0.001**
Handgrip strength (kg)	23.12 ± 3.35	22.71 ± 3.04	14.48 ± 2.74	1.000	**<0.001**	**<0.001**
Education (years)	11.91 ± 3.63	9.85 ± 2.14	8.97 ± 2.28	**0.001**	**<0.001**	0.591
IPAQ-SF (MET min/week)	2336.22 ± 2039.29	2536.36 ± 2558.40	1957.56 ± 1874.85	1.000	1.000	0.740

All data represent the mean ± standard deviation; Cog, cognitive function; MS, muscle strength; BMI, body mass index; MMSE, minimental state examination; IPAQ-SF, international physical activity questionnaire-short form; MET, metabolic equivalents; Boldface indicates significant differences, *p* < 0.0167.

The participants were grouped based on their performance in the Korean Mini-Mental Status Examination (MMSE), using a cut-off score of 24 points (26), to assess Cog. In addition, PF and MS measurements were used to categorize the groups according to the diagnostic criteria for sarcopenia established by the Asian Working Group (9). The classification of the participants’ Cog, PF, and MS declines were defined as follows: group without signs of declines in Cog and PF (Cog+PF+), group with Cog decline but no signs of PF decline (Cog−PF+), and group with signs of declines in Cog and PF (Cog−PF−), group without signs of declines in Cog and MS (Cog+MS+), group with Cog decline but no signs of MS decline (Cog−MS+), and group with signs of declines in Cog and MS (Cog−MS−). The experimental protocols were approved by the institutional review board (IRB number: 2–104,709–AB–N–01–201,808–HR–023–02), and all participants provided written informed consent before participating in this study.

### 2.2 Test procedures

The experimental process involved obtaining informed consent, measuring physical characteristics, conducting the International Physical Activity Questionnaire-Short Form (IPAQ-SF), MMSE, performing PF and MS assessments, and gait analysis. The IPAQ-SF was used to assess physical activity levels, and the frequency of activity (days per week) was assessed and the corresponding metabolic equivalent (MET) was calculated (MET per week) based on the results of the questionnaire (27). The MMSE comprises assessments of orientation, registration, attention and calculation, recall, language, and visual–spatial abilities. Out of 30 points, scores of 24 or above are considered normal, scores between 20 and 23 indicate mild cognitive impairment, and scores of 19 or below are categorized as severe cognitive impairment (28).

All participants wore comfortable and lightweight attire during the experiments. To acclimatize to the experimental environment, PF, MS, and gait assessments were conducted after warming up, by walking and stretching, for approximately 5 min. PF was assessed by using the FSTS test, one of the physical performance tests (9). HGS was assessed for MS using an isometric digital hand dynamometer (TKK 5401 Grip-D; Takei Scientific Co., Ltd., Tokyo, Japan), with the maximum value recorded after two measurements for each hand (9). The FSTS test and HGS have excellent test–retest reliability in healthy older adults (intraclass correlation coefficient (ICC) range: 0.914–0.933 and 0.95–0.96) (29, 30).

**Figure 2:**
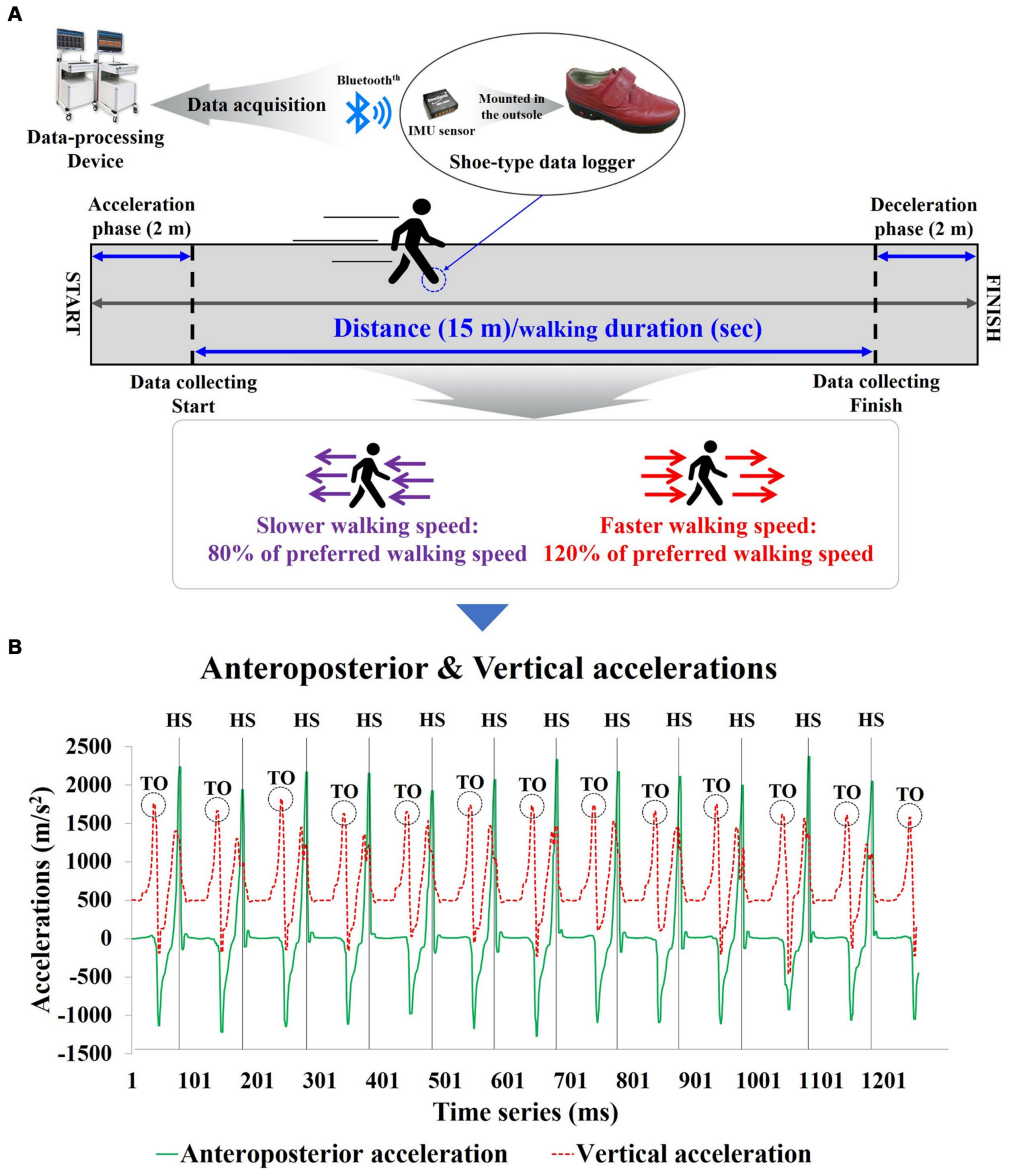
Shoe-type inertial measurement units-based gait analysis system for the study; **(A)** Data Collection and analysis phase; **(B)** Detection of gait events with the shoe-type inertial measurement unit (IMU) system. Data is collected at 100 Hz. HS, heel stride; TO, toe-off.

For gait assessment, all participants walked along a 19-meter straight path at their self-selected, comfortable, and usual walking pace to measure their preferred walking speed (PWS). Additionally, a metronome measurement of PWS was taken simultaneously to quantify slower walking speed (SWS) and faster walking speed (FWS). The measurements for SWS (80% of PWS) and FWS (120% of PWS) were calculated based on the participants’ PWS. Participants practiced 3–5 repetitions with the metronome to adapt to the quantified speeds. After the adaptation phase, the metronome was excluded and gait assessment was performed. All gait assessment was calculated by dividing the time taken to traverse a 15-meter distance (excluding the initial 2 meters of acceleration and final 2 meters of deceleration) (Figure 2) (31).

### 2.3 Instrumentation

The gait analysis equipment used in the study included a shoe-mounted data collection device equipped with inertial measurement unit (IMU) sensors (Smart Balance^Ⓡ^ SB-1, JEIOS, Korea) and a gait analysis system (DynaStab™, JEIOS, Korea) (Figure 2). The shoe-mounted data collection device consists of an inertial sensor (IMU-3000™, Invense, United States) capable of measuring triaxial accelerations (up to ±6 g) and triaxial angular velocities (up to ±500°s^−1^) along three orthogonal axes (32, 33). The IMU sensors were embedded in the outsoles of both shoes, and the measured data were transmitted to the gait analysis system using Bluetooth^Ⓡ^ communication.

### 2.4 Gait data collection and processing

**Figure 3:**
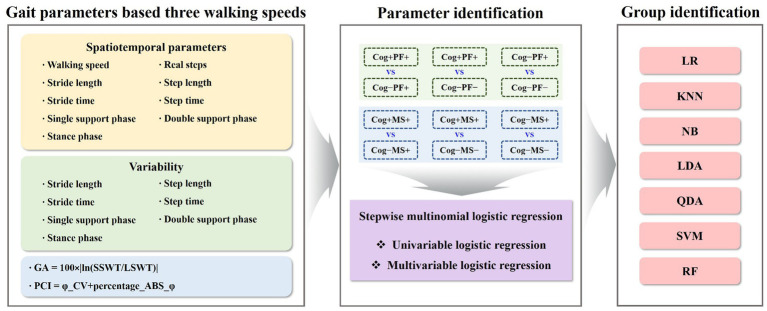
Gait parameters-based machine-learning analysis for identifying groups. GA, gait asymmetry; PCI, phase coordination index; Cog, cognitive function; PF, physical function; MS, muscle strength; LR, logistic regression; KNN, k − nearest neighbors; NB, naїve bayes; LDA, linear discriminant analysis; QDA, quadratic discriminant analysis; SVM, support vector machine; RF, random forest.

Gait data were collected at a rate of 100 Hz; then, they were filtered using a second-order Butterworth low-pass filter with a cutoff frequency of 10 Hz (32, 33). We calculated spatiotemporal parameters, including walking speed, stride length, step length, real steps, stride time, step time, single support, double support, and stance phases. For GV, spatiotemporal parameters were quantified as the coefficient of variance (CV; standard deviation/mean × 100) to assess their variability. Gait asymmetry (GA) was calculated by comparing the swing time of one leg with that of the other (34). The phase coordination index (PCI) is the sum of the two percentile values that reflect the accuracy and consistency of bilateral coordination during the gait task (34) (Figure 3).

### 2.5 Statistical analysis

The Shapiro–Wilk test revealed that the data followed normal distribution. A oneway analysis of variance (ANOVA) with the Bonferroni post-hoc test was performed to analyze the mean, standard deviation (SD), and 95% CI (min to max) for the groups (Cog+PF+, Cog−PF+, and Cog−PF− and Cog+MS+, Cog−MS+, and Cog−MS−) with the statistical significance levels set at 0.0167 (0.05/3). An ICC analysis (2,1) was conducted to confirm the reliability between the relatively calculated and executed SWS and FWS values based on the participants’ PWS. The limits of agreement (LoAs) between the measured and estimated walking speeds were calculated using the Bland–Altman plot (35).

Univariate and multivariate logistic regression analyses using stepwise regression were performed to identify the best combination of gait characteristics for the optimal classification of groups. Covariates included age, height, body mass index (BMI), and years of education. Before additional analyses, all variables were Z-normalized (value–mean/standard deviation). Stepwise binary logistic regression analysis was performed to identify the classifier variables that distinguish the groups. The classifier variables were expressed as the odds ratios (OR) with 95% CI.

Moreover, to distinguish each group, the optimal cutoff values of the gait features were identified using receiver operating characteristic (ROC) curves. This cut-off value was defined as the point on the ROC curve closest to the upper-left corner of the graph. Areas under the curve (AUC) of the ROC curves were calculated to determine the classification accuracy of each group. An AUC > 0.9 has high accuracy, whereas AUCs of 0.7–0.9 and 0.5–0.7 indicate moderate and low accuracies, respectively (36). All statistical analyses were performed using the SPSS 21.0 (IBM Corp., Armonk, NY). Statistical significance was set at *p* < 0.05.

To solve the group classification problems, the study investigated seven traditional ML techniques: Logistic Regression (LR) (37), KNearest Neighbors (KNN) (38), Naїve Bayes (NB) (39), Linear Discriminant Analysis (LDA) (40), Quadratic Discriminant Analysis (QDA) (40), SVM (41), and RF (42). The model parameters of the classifiers were estimated using a grid search. The estimated model parameters for the six cases are presented in Tables 3, 4. In the analysis, we evaluated the accuracy, recall, precision, and F1 score using 5-fold cross-validation. However, the collected participants’ datasets in the group classification problem were imbalanced. To address this imbalance, we employed a random oversampling approach (43) (Figure 3).

**Table 3:** Model parameters of the 7 classifiers estimated by the grid search based on Cog and PF declines group classification.

ML techniques	Cog + PF+ vs. Cog − PF+ (w/96 Features)	Cog + PF+ vs. Cog − PF+ (w/3 Features)	Cog + PF+ vs. Cog − PF− (w/96 Features)	Cog + PF+ vs. Cog − PF− (w/3 Features)	Cog − PF+ vs. Cog − PF− (w/96 Features)	Cog − PF+ vs. Cog − PF− (w/1 Feature)
LR	C = 0.01	C = 0.001	C = 1.0	C = 0.01	C = 0.01	C = 0.001
KNN	k = 2	k = 9	k = 3	k = 7	k = 2	k = 8
NB	−	−	−	−	−	−
LDA	n_components = 1	n_components = 1	n_components = 1	n_components = 1	n_components = 1	n_components = 1
QDA	reg_param = 0.001	reg_param = 0.5	reg_param = 0.5	reg_param = 0.2	reg_param = 0.001	reg_param = 0.001
SVM	C = 0.508, gamma = 10.0, kernel = rbf	C = 0.508, gamma = 1000.0, kernel = rbf	C = 1.968, gamma = 0.01, kernel = rbf	C = 1719.072 gamma = 0.001, kernel = rbf	C = 1.968, gamma = 0.001, kernel = rbf	C = 114.505, gamma = 1.0, kernel = rbf
RF	max_depth = 20, n_estimators = 1,000	max_depth = 15, n_estimators = 750	max_depth = 10, n_estimators = 1,000	max_depth = 10, n_estimators = 1,000	max_depth = 20, n_estimators = 500	max_depth = 30, n_estimators = 1,250

ML, Machine learning; Cog, cognitive function; PF, physical function; LR, logistic regression, ‘C’ is the inverse of regularization strength; KNN, k − nearest neighbors, ‘k’ is the number of neighbors; NB, naїve bayes; LDA, linear discriminant analysis, ‘n_components’ is the number of components; QDA, quadratic discriminant analysis, ‘reg_param’ is the regularization of the per − class covariance; SVM, support vector machine, ‘C’ is the regularization parameter and ‘gamma’ is the kernel coefficient; RF, random forest, ‘n_estimators’ is the number of trees in the forest and ‘max_depth’ is the maximum depth of the tree.

**Table 4:** Model parameters of the 7 classifiers estimated by the grid search based on Cog and MS declines group classification.

ML techniques	Cog + MS+ vs. Cog − MS+ (w/96 Features)	Cog + MS+ vs. Cog − MS+ (w/1 Feature)	Cog + MS+ vs. Cog − MS− (w/96 Features)	Cog + MS+ vs. Cog − MS− (w/2 Features)	Cog − MS+ vs. Cog − MS− (w/96 Features)	Cog − MS+ vs. Cog − MS− (w/1 Feature)
LR	C = 1000.0	C = 0.1	C = 1000.0	C = 0.01	C = 0.001	C = 1.0
KNN	k = 3	k = 2	k = 3	k = 2	k = 2	k = 9
NB	**−**	**−**	**−**	**−**	**−**	**−**
LDA	n_components = 1	n_components = 1	n_components = 1	n_components = 1	n_components = 1	n_components = 1
QDA	reg_param = 0.001	reg_param = 0.4	reg_param = 0.001	reg_param = 0.4	reg_param = 0.001	reg_param = 0.4
SVM	C = 1e**−**05, gamma = 1.0, kernel = rbf	C = 1e**−**05, gamma = 100000.0, kernel = rbf	C = 1.968, gamma = 1.0, kernel = rbf	C = 1.968, gamma = 100000.0, kernel = rbf	C = 1e**−**05, gamma = 10.0, kernel = rbf	C = 6660.846, gamma = 0.001, kernel = rbf
RF	max_depth = 10, n_estimators = 500	max_depth = 10, n_estimators = 500	max_depth = 10, n_estimators = 750	max_depth = 25, n_estimators = 500	max_depth = 25, n_estimators = 500	max_depth = 10, n_estimators = 500

ML, Machine learning; Cog, cognitive function; MS, muscle strength; LR, logistic regression, ‘C’ is the inverse of regularization strength; KNN, k − nearest neighbors, ‘k’ is the number of neighbors; NB, naїve bayes; LDA, linear discriminant analysis, ‘n_components’ is the number of components; QDA, quadratic discriminant analysis, ‘reg_param’ is the regularization of the per − class covariance; SVM, support vector machine, ‘C’ is the regularization parameter and ‘gamma’ is the kernel coefficient; RF, random forest, ‘n_estimators’ is the number of trees in the forest and ‘max_depth’ is the maximum depth of the tree.

## 3 Results

### 3.1 Reliability of estimation and measurement speeds

**Figure 4:**
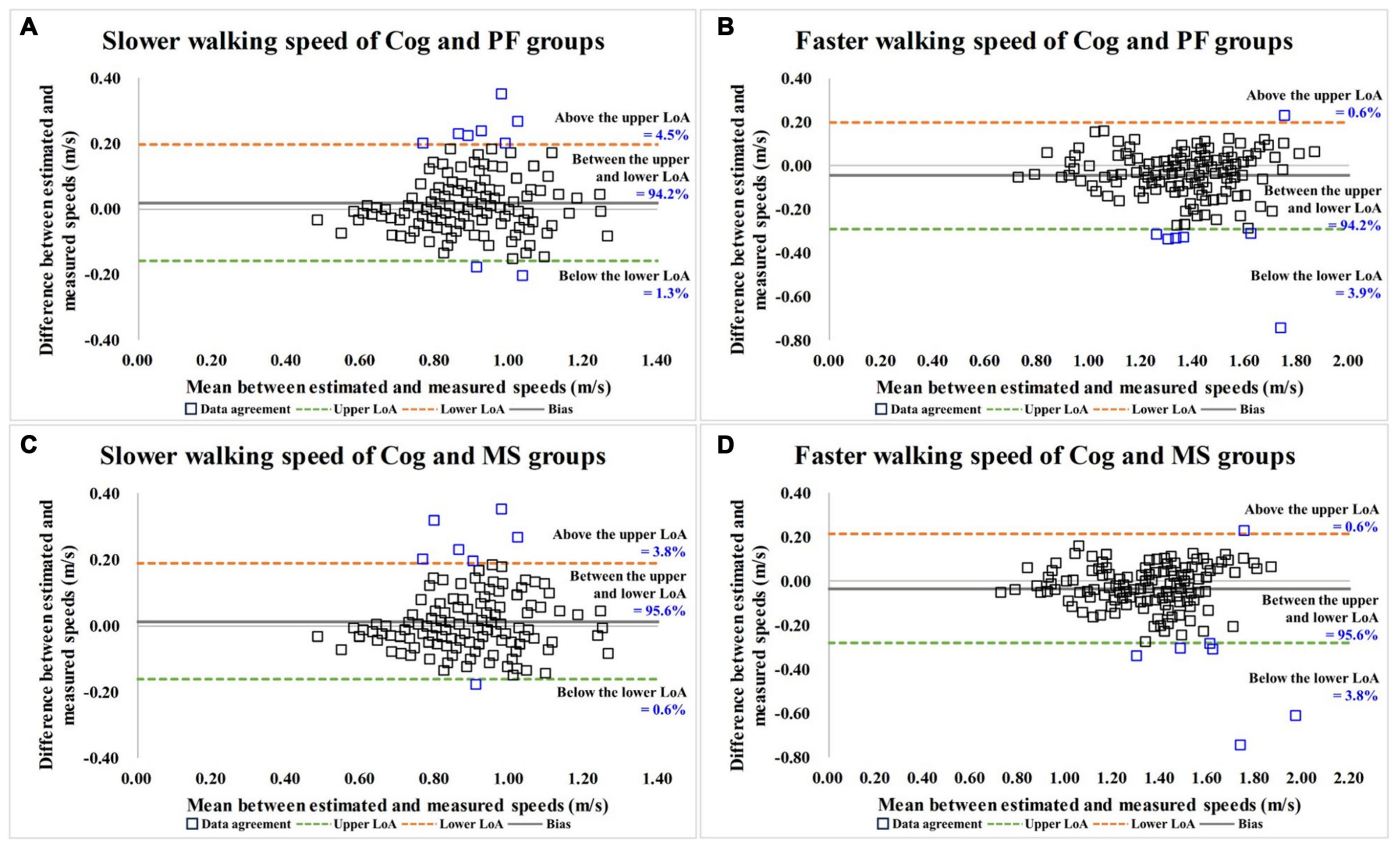
Bland–Altman plots for data agreement between the estimated and measured over-ground walking speeds. **(A,B)** are the slower and faster walking speed results for Cog and PF groups; **(C,D)** are the slower and faster walking speed results for Cog and MS groups. Cog, cognitive function; PF, physical function; MS, muscle strength; LoA, limits of agreement.

As shown in Table 5 and Figure 4, the degrees of agreement at SWS and FWS for participants in the Cog and PF groups were 89.5 and 91.2%, respectively. For participants in the Cog and MS groups, the degree of agreement at SWS and FWS were 90.2 and 91.8%, respectively.

**Table 5:** Reliability of the results for slower and faster walking speeds.

	Cog and PF groups (*n* = 154)	Cog and MS groups (*n* = 158)
SWS		
Estimated/measured (m/s)	0.88/0.90	0.88/0.89
ICC (2,1)	0.895	0.902
*p*-value	<0.001	<0.001
FWS		
Estimated/measured (m/s)	1.39/1.35	1.37/1.34
ICC (2,1)	0.912	0.918
*p*-value	<0.001	<0.001

All data represent the mean ± standard deviation; Cog, cognitive function; PF, physical function; MS, muscle strength; SWS, slower walking speed; FWS, faster walking speed; ICC, intraclass correlation coefficient; significant difference, *p* < 0.05.

### 3.2 Classification by Cog and PF declines using feature selection through stepwise regression

Table 6 presents the results of the stepwise regression procedure for the classification of Cog+PF+ and Cog−PF+, Cog+PF+ and Cog−PF−, and Cog−PF+ and Cog−PF−. In the classification of Cog+PF+ and Cog−PF+, the selected gait features included the CV of the left double support phase at SWS (Cutoff value: 11.90%; AUC: 0.345, *p* = 0.006; sensitivity: 0.446; specificity: 0.452), the CV of the left double support phase at FWS (Cutoff value: 10.44%; AUC: 0.382, *p* = 0.035; sensitivity: 0.405; specificity: 0.429), and the right double support phase at SWS (Cutoff value: 19.26%; AUC: 0.593, *p* = 0.097; sensitivity: 0.595; specificity: 0.571). In the classification of Cog+PF+ and Cog−PF−, the selected gait features included the walking speed (Cutoff value: 1.38 m/s; AUC: 0.813, *p* < 0.001; sensitivity: 0.784; specificity: 0.737), CV of the left double support phase at FWS (Cutoff value: 2.09%; AUC: 0.293, p < 0.001; sensitivity: 0.378; specificity: 0.368), and the right double support phase at SWS (Cutoff value: 19.69%; AUC: 0.479, *p* = 0.715; sensitivity: 0.527; specificity: 0.526). In the classification of Cog−PF+ and Cog−PF−, the selected gait feature was the walking speed at PWS (Cutoff value: 1.01 m/s; AUC: 0.742, p < 0.001; sensitivity: 0.786; specificity: 0.632).

**Table 6:** Feature selection of three speed-based gait characteristics using stepwise regression procedure by Cog and PF classification problems.

Variables	Estimate (SE)	OR (95% CI)	*p* value	R_N_^2^
Cog + PF+ and Cog–PF+				
CV of left double support phase (slower) (11.90%)	0.795 (0.273)	2.213 (1.295–3.783)	0.004	0.481
CV of left double support phase (faster) (10.44%)	0.668 (0.316)	1.950 (1.049–3.624)	0.035	
Right double support phase (slower) (19.26%)	0.776 (0.299)	2.173 (1.208–3.907)	0.010	
Cog + PF+ and Cog–PF–				
Walking speed (faster) (1.38 m/s)	−1.575 (0.547)	0.207 (0.071–0.605)	0.004	0.810
CV of left double support phase (faster) (2.09%)	2.965 (0.797)	19.390 (4.067–92.445)	<0.001	
Right double support phase (slower) (19.69%)	0.984 (0.433)	2.674 (1.143–6.253)	0.023	
Cog–PF+ and Cog–PF–				
Walking speed (preferred) (1.01 m/s)	−0.827 (0.323)	0.437 (0.232–0.825)	0.011	0.296

Model was adjusted for age, height, body mass index, and education. Cog, cognitive function; PF, physical function; SE, standard error; OR, odds ratio: 95% CI, 95% confidence interval; R_N_^2^ is the fit statistic for the Nagelkerke Model; CV, coefficient of variance; Boldface indicates significant differences, *p* < 0.05.

**Figure 5:**
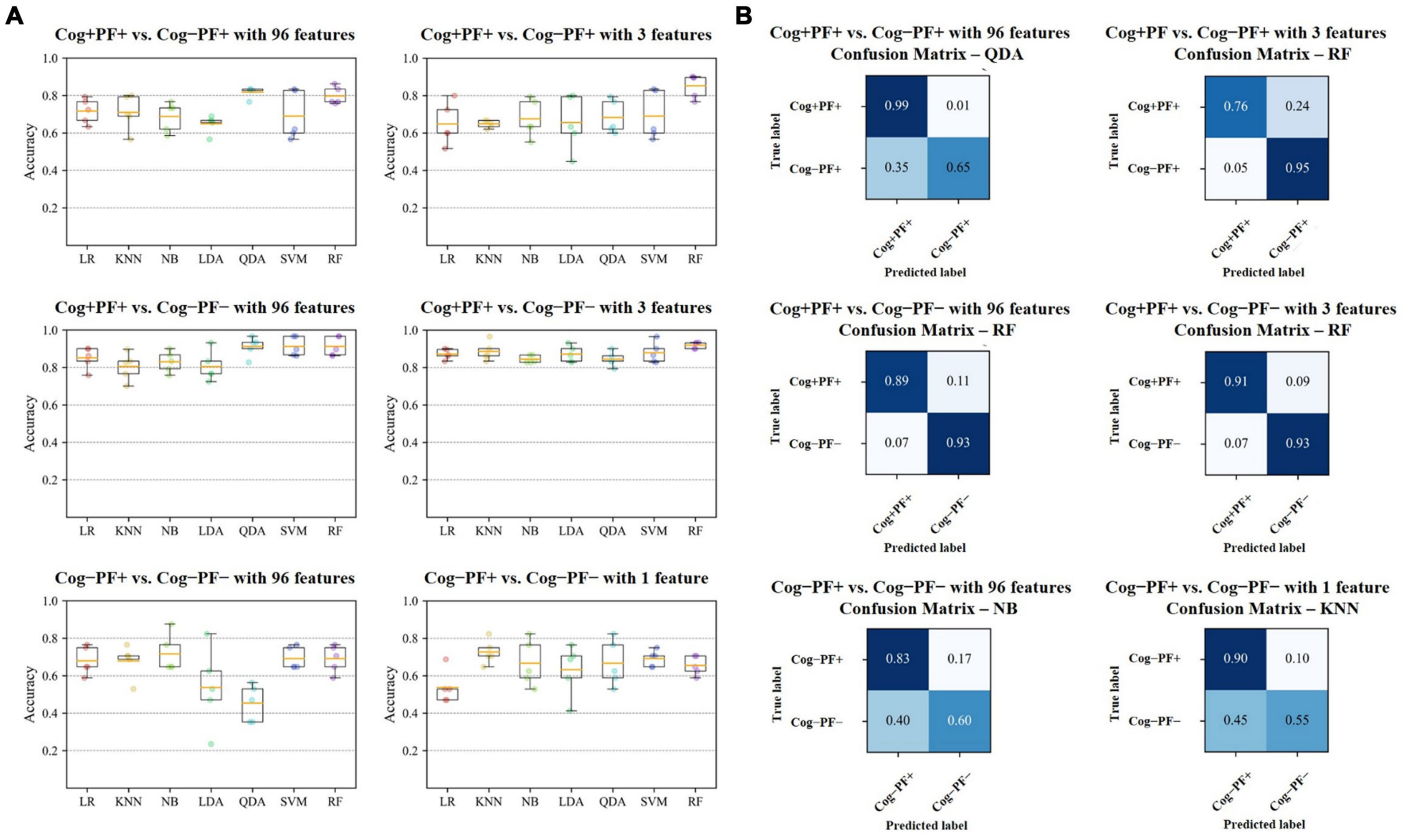
Results of Cog and PF classification problems **(A)** Accuracy of the seven classifiers. The orange line in the box plot shows the mean values. **(B)** Confusion matrices of the six cases. Cog, cognitive function; PF, physical function; LR, Logistic Regression; KNN, K-Nearest Neighbors; NB, Naїve Bayes; LDA, Linear Discriminant Analysis; QDA, Quadratic Discriminant Analysis; SVM, Support Vector Machine; RF, Random Forest.

We investigated the accuracy and confusion matrix to estimate the binary classification problems (Cog and PF classification problems); the recall, precision, and F1 score results are listed in Supplementary Table S1. This study addresses three classification problems involving two different feature sets (using all the features and those selected through stepwise regression). We organized the results for the following six cases: (1) Cog+PF+ vs. Cog−PF+ with 96 features (Cog+PF+ vs. Cog−PF + _96), (2) Cog+PF+ vs. Cog−PF+ with 3 features selected through stepwise regression (Cog+PF+ vs. Cog−PF + _3), (3) Cog+PF+ vs. Cog−PF− with 96 features (Cog+PF+ vs. Cog−PF − _96), (4) Cog+PF+ vs. Cog−PF− with 3 features selected through stepwise regression (Cog+PF+ vs. Cog−PF −_3), (5) Cog−PF+ vs. Cog−PF− with 96 features (Cog−PF+ vs. Cog−PF − _96), and (6) Cog−PF+ vs. Cog−PF− with 1 feature selected through stepwise regression (Cog−PF+ vs. Cog−PF − _1). Table 7 shows the average accuracy and standard deviation (SD), calculated through 5fold cross-validation, and the accuracy box plots of the Cog and PF classification problems in the six cases are shown in Figure 5A.

**Table 7:** Accuracies of 7 classifiers from 5-fold cross validation by Cog and PF classification problems.

	ML techniques	Cog + PF+ vs. Cog–PF+ (w/96 Features)	Cog + PF+ vs. Cog–PF+ (w/3 Features)	Cog + PF+ vs. Cog–PF– (w/96 Features)	Cog + PF+ vs. Cog–PF– (w/3 Features)	Cog–PF+ vs. Cog–PF– (w/96 Features)	Cog–PF+ vs. Cog–PF– (w/1 Feature)
Accuracy	LR	71.7 ± 6.7	64.8 ± 11.2	85.1 ± 5.9	87.2 ± 2.7	67.9 ± 7.5^*^	53.8 ± 8.9^*^
	KNN	71.0 ± 9.5	64.9 ± 2.1	80.5 ± 7.4^*^	88.6 ± 5.1^*^	67.9 ± 8.8	**72.6 ± 6.5**
	NB	68.8 ± 7.9	67.6 ± 10.1	83.0 ± 5.6	84.4 ± 2.0	**71.6 ± 10.2**	66.6 ± 12.3
	LDA	64.9 ± 4.8	65.5 ± 14.7	80.4 ± 8.1	87.2 ± 4.4	53.7 ± 21.5	63.2 ± 13.8
	QDA	81.8 ± 2.9^*^	68.3 ± 9.0^*^	91.2 ± 5.3^*^	84.4 ± 4.0^*^	45.4 ± 9.8	66.6 ± 12.3
	SVM	69.0 ± 13.0	69.0 ± 13.0	**91.2 ± 5.2**	87.9 ± 5.7	69.1 ± 6.1	69.1 ± 4.4
	RF	79.7 ± 4.7	**85.2 ± 6.4**	**91.2 ± 5.2**	**91.9 ± 1.7**	69.1 ± 7.4	65.4 ± 5.1

Precision, recall, and F1 score are represented as mean (%) ± standard deviation (%). ML, machine learning; Cog, cognitive function; PF, physical function; LR, logistic regression; KNN, knearest neighbors; NB, naїve bayes; LDA, linear discriminant analysis; QDA, quadratic discriminant analysis; SVM, support vector machine; RF, random forest. ^*^ Denotes a significant difference. Boldface indicates the highest accuracy.

In the Cog+PF+ and Cog−PF+ classification problem, LDA showed the lowest accuracy (64.9% ± 4.8% SD) and QDA showed the highest accuracy (81.8% ± 2.9% SD) for Cog+PF+ vs. Cog−PF + _96. Stepwise regression was applied to the results of Cog+PF+ vs. Cog−PF + _3 to reduce the feature size; LR showed the lowest accuracy (64.8% ± 11.2% SD) and RF showed the highest accuracy (85.2% ± 6.4% SD). In the Cog+PF+ and Cog−PF+ classification problem, RF showed that the reduction of feature sets by stepwise regression exhibited no significant differences among cases with all 96 features, and the ML analysis results of cases with three features outperformed those of cases with 96 features.

In the Cog+PF+ and Cog−PF− classification problem, LDA had the lowest accuracy for Cog+PF+ vs. Cog−PF − _96 (80.4% ± 8.1% SD), whereas QDA, SVM, and RF had the highest accuracy (QDA: 91.2% ± 5.3%, SVM: 91.2% ± 5.2%, RF: 91.2% ± 5.2%). QDA had the lowest accuracy for Cog+PF+ vs. Cog−PF − _3 (84.4% ± 4.0% SD), whereas RF had the highest accuracy (91.9% ± 1.7% SD). Overall, the difference in accuracy between Cog+PF+ vs. Cog−PF − _96 and between Cog+PF+ vs. Cog−PF − _3 was not large, which means that most of the classifiers effectively distinguish between Cog+PF+ and Cog−PF−. In addition, most classifiers showed similarly high accuracy performance after reducing the number of features, except for KNN and QDA. In the Cog−PF+ and Cog−PF− classification problem, QDA had the lowest accuracy for Cog−PF+ vs. Cog−PF − _96 (45.4% ± 9.8% SD), whereas NB had the highest accuracy (71.6% ± 10.2%). LR had the lowest accuracy for Cog−PF+ vs. Cog−PF − _1 (53.8% ± 8.9% SD), whereas KNN had the highest accuracy (72.6% ± 6.5% SD). The confusion matrices for the six cases are shown in Figure 5B. In the Cog+PF+ and Cog−PF− classification problems, RF exhibited high accuracy performance, which was also confirmed using the confusion matrix.

### 3.3 Classification by Cog and MS declines using feature selection through stepwise regression

The results of the stepwise regression procedure for the classification of Cog+MS+ and Cog−MS+, Cog+MS+ and Cog−MS−, and Cog-MS+ and Cog−MS− are shown in Table 8. In the classification of Cog+MS+ and Cog−MS+, the selected gait feature was the CV of the left double support phase at SWS (Cutoff value: 12.08%; AUC: 0.341, *p* = 0.003; sensitivity: 0.385; specificity: 0.375). In the classification of Cog+MS+ and Cog−MS−, the selected gait features were the left step time at PWS (Cutoff value: 51.50 cm; AUC: 0.413, *p* = 0.151; sensitivity: 0.359; specificity: 0.500) and GA at SWS (Cutoff value: 2.18; AUC: 0.498, *p* = 0.057; sensitivity: 0.513; specificity: 0.500). In the classification of Cog−MS+ and Cog−MS−, the selected gait feature was the CV of the right single-support phase at PWS (Cutoff value: 3.02; AUC: 0.268, *p* < 0.001; sensitivity: 0.292; specificity: 0.281).

**Table 8:** Feature selection of three speed-based gait characteristics using stepwise regression procedure by Cog and MS classification problems.

Variables	Estimate (SE)	OR (95% CI)	*p* value	R_N_^2^
Cog + MS+ and Cog–MS+				
CV of left double support phase (slower) (12.08%)	0.481 (0.242)	1.618 (1.008–2.599)	0.046	0.184
Cog + MS+ and Cog–MS–				
Left step time (preferred) (51.50 cm)	0.731 (0.317)	2.078 (1.115–3.872)	0.025	0.558
GA (slower) (2.18)	−0.930 (0.415)	0.395 (0.175–0.890)	0.025	
Cog–MS+ and Cog–MS–				
CV of right single support phase (preferred) (3.02%)	1.234 (0.524)	3.433 (1.229–9.589)	0.019	0.442

Model was adjusted for age, height, body mass index, and education. Cog, cognitive function; MS, muscle strength; SE, standard error; OR, odds ratio: 95% CI, 95% confidence interval; R_N_^2^ is the fit statistic for the Nagelkerke Model; CV, coefficient of variance; Boldface indicates significant differences, *p* < 0.05.

**Figure 6:**
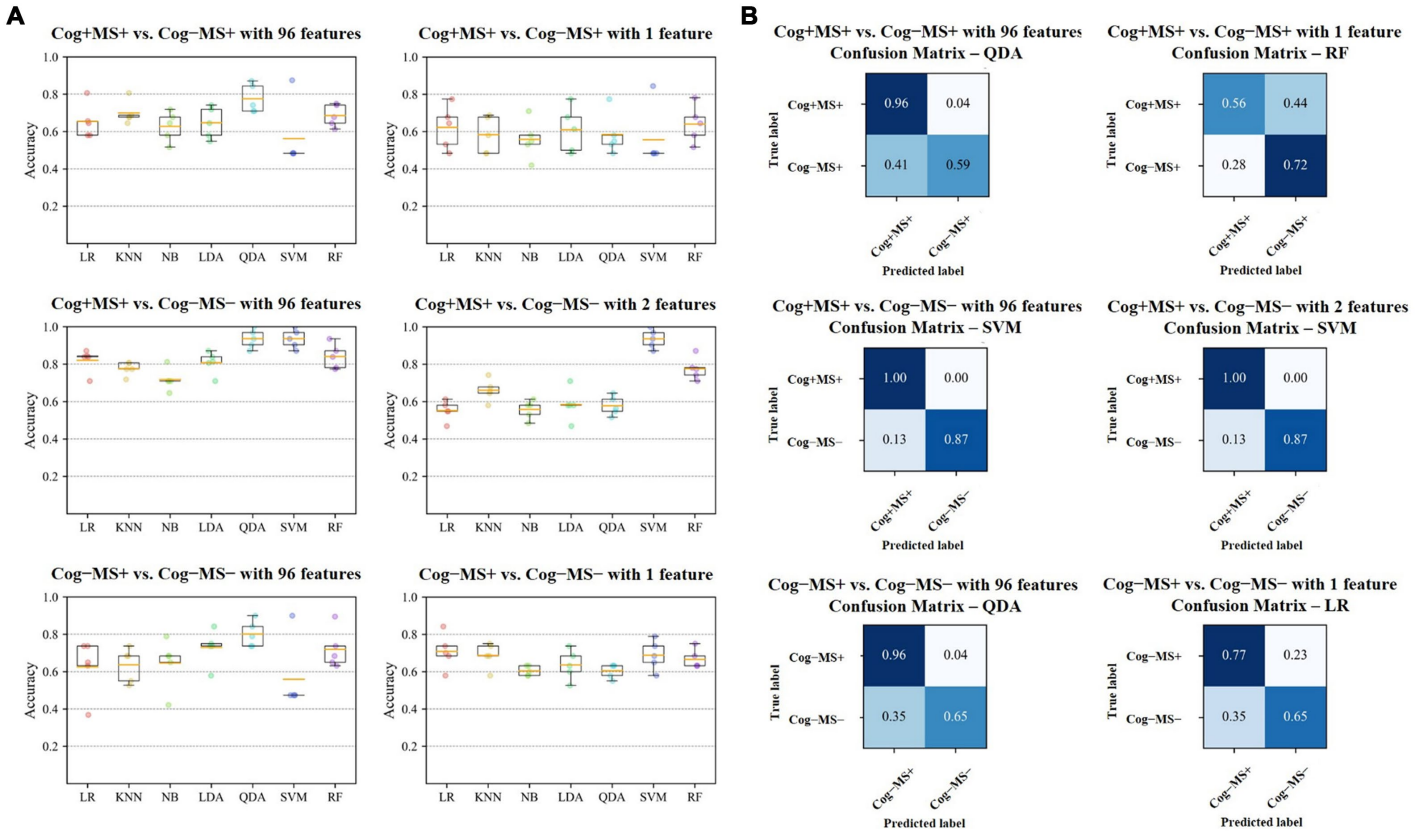
Results of Cog and MS classification problems **(A)** Accuracy of the seven classifiers. The orange line in the box plot shows the mean values. **(B)** Confusion matrices of the six cases. Cog, cognitive function; MS, muscle strength; LR, Logistic Regression; KNN, K-Nearest Neighbors; NB, Naїve Bayes; LDA, Linear Discriminant Analysis; QDA, Quadratic Discriminant Analysis; SVM, Support Vector Machine; RF, Random Forest.

We investigated the accuracy and confusion matrix to estimate the binary classification problems (Cog and MS classification problems); the recall, precision, and F1 score results are listed in Supplementary Table S2. This study addresses three classification problems involving two different feature sets (using all the features and those selected through stepwise regression). We organized the results for the following six cases: (1) Cog+MS+ vs. Cog−MS+ with 96 features (Cog+MS+ vs. Cog−MS + _96), (2) Cog+MS+ vs. Cog−MS+ with 1 feature selected through stepwise regression (Cog+MS+ vs. Cog−MS + _1), (3) Cog+MS+ vs. Cog−MS− with 96 features (Cog+MS+ vs. Cog−MS − _96), (4) Cog+MS+ vs. Cog−MS− with 2 features selected through stepwise regression (Cog+MS+ vs. Cog−MS − _2), (5) Cog−MS+ vs. Cog−MS− with 96 features (Cog−MS+ vs. Cog−MS − _96), and (6) Cog−MS+ vs. Cog−MS− with 1 feature (Cog−MS+ vs. Cog−MS − _1) selected through stepwise regression. Table 9 indicates the average accuracy and standard deviation (SD) calculated through 5-fold cross-validation, and Figure 6A shows the accuracy box plots of the Cog and MS classification problems for the six cases.

**Table 9:** Accuracies of 7 classifiers from 5-fold cross validation by Cog and MS classification problems.

	ML techniques	Cog + MS+ vs. Cog–MS+ (w/96 Features)	Cog + MS+ vs. Cog–MS+ (w/1 Feature)	Cog + MS+ vs. Cog–MS– (w/96 Features)	Cog + MS+ vs. Cog–MS– (w/2 Features)	Cog–MS+ vs. Cog–MS– (w/96 Features)	Cog–MS+ vs. Cog–MS– (w/1 Feature)
Accuracy	LR	65.4 ± 9.2	62.2 ± 11.6	82.0 ± 6.3^*^	55.2 ± 5.4^*^	62.5 ± 15.1	**70.8 ± 9.5**
	KNN	69.9 ± 6.2	58.3 ± 9.9	77.6 ± 3.6^*^	66.0 ± 5.8^*^	63.6 ± 9.3	68.7 ± 6.7
	NB	62.8 ± 8.0	55.8 ± 10.4	71.7 ± 6.0^*^	55.8 ± 5.1^*^	64.6 ± 13.6	60.4 ± 2.6
	LDA	64.7 ± 8.4	61.0 ± 12.2	80.8 ± 6.0^*^	58.4 ± 8.5^*^	72.9 ± 9.5^*^	63.6 ± 8.0^*^
	QDA	**77.5 ± 7.7**	58.4 ± 11.2	93.6 ± 5.1^*^	57.7 ± 5.2^*^	80.1 ± 7.0^*^	60.5 ± 3.8^*^
	SVM	56.2 ± 17.5	55.6 ± 16.1	**93.6 ± 5.1**	**93.6 ± 5.1**	55.9 ± 19.1	68.8 ± 8.1
	RF	68.5 ± 6.0	**64.0 ± 10.0**	84.0 ± 6.7	77.6 ± 6.0	71.9 ± 10.6	66.6 ± 5.2

Precision, recall, and F1 score are represented as mean (%) ± standard deviation (%). Cog, cognitive function; MS, muscle strength; LR, logistic regression; KNN, knearest neighbors; NB, naїve bayes; LDA, linear discriminant analysis; QDA, quadratic discriminant analysis; SVM, support vector machine; RF, random forest. ^*^Denotes a significant difference. Boldface indicates the highest accuracy.

In the Cog+MS+ and Cog−MS+ classification problem, SVM showed the lowest accuracy (56.2% ± 17.5% SD) and QDA showed the highest accuracy (77.5% ± 7.7% SD) for Cog+MS+ vs. Cog−MS+ _96. Stepwise regression was applied to the results of Cog+MS+ vs. Cog−MS + _1 to reduce the feature size; SVM showed the lowest accuracy (55.6% ± 16.1% SD) and RF showed the highest accuracy (64.0% ± 10.0% SD).

In the Cog+MS+ and Cog−MS− classification problem, NB had the lowest accuracy for Cog+MS+ vs. Cog−MS − _96 (71.7% ± 6.0% SD), whereas QDA and SVM had the highest accuracy (93.6% ± 5.1%). LR had the lowest accuracy for Cog+MS+ vs. Cog−MS − _2 (55.2% ± 5.4% SD), whereas SVM had the highest accuracy (93.6% ± 5.1% SD). In addition, the reduction of feature sets by stepwise regression resulted in a significant difference between the cases where all 96 features were used and those where only selected features were used, except for SVM and RF. The results of the ML analysis of 96 features outperformed those of the ML analysis of 2 features.

In the Cog−MS+ and Cog−MS− classification problem, SVM had the lowest accuracy for Cog−MS+ vs. Cog−MS − _96 (55.9% ± 19.1% SD), whereas QDA had the highest accuracy (80.1% ± 7.0%). NB had the lowest accuracy for Cog−MS+ vs. Cog−MS − _1 (60.4% ± 2.6% SD), whereas LR had the highest accuracy (70.8% ± 9.5% SD). Figure 6B shows the confusion matrices for the six cases. In the Cog+MS+ and Cog−MS− classification problem, SVM showed high accuracy performance, which was confirmed using the confusion matrix.

## 4 Discussion

### 4.1 Classification by Cog and PF declines using feature selection through stepwise regression

In this study’s main findings for classification by Cog and PF declines, the three selected features that were most relevant for the classification of Cog+PF+ and Cog–PF+ were the CV of the left double support phase at SWS and at FWS and the right double support phase at SWS. The ML approach showed that QDA had 81.8% accuracy for Cog+PF+ vs. Cog–PF+ _96, and RF had 85.2% accuracy for Cog+PF+ vs. Cog–PF+ _3. In the classification between Cog+PF+ and Cog–PF–, the three selected features that were most relevant were gait walking speed, CV of the left double support phase at FWS, and the right double support phase at SWS. The ML approach showed that RF solved the Cog+PF+ and Cog–PF– classification problems with 91.2 and 91.9% accuracies for Cog+PF+ vs. Cog–PF–_96 and Cog+PF+ vs. Cog–PF–_3, respectively. In the classification between Cog–PF+ and Cog–PF–, the selected feature that was most relevant was walking speed at PWS. The ML approach showed that NB had 71.6% accuracy for Cog–PF+ vs. Cog–PF– _96 and KNN had 72.6% accuracy for Cog–PF+ vs. Cog–PF– _1. The findings are discussed in detail below.

Regarding the classification of Cog+PF+ and Cog–PF+, the CV of the left double support phase at SWS and at FWS and the right double support phase at SWS were selected through stepwise regression to obtain the sensitive cutoff values in the ROC analysis. The double-support phase of gait is the only period in which both feet are in contact with the ground, and this phase is more variable with poor balance (44–46). In previous studies, double support time variability was associated with a greater number of sensorimotor (46) and cognitive domains than other variability measures (47), and these variations indicated an increased risk of falls (48). Jayakody et al. (44) reported that higher double-support time variability is associated with memory decline. The hippocampus and parahippocampal gyrus are the primary areas of memory and spatial navigation (49), and these appear to be involved in human balance control (50). Therefore, age-related changes in the hippocampus are likely to lead to a greater double-support phase, CV of the double-support phase, and memory impairment (44).

For classification between Cog+PF+ and Cog−PF−, walking speed, CV of the left double support phase at FWS, and the right double support phase at SWS were selected via stepwise regression to obtain the sensitive cut-off values in the ROC analysis. In particular, CV of the left double support phase at FWS was shown 19.39 times and walking speed at FWS was shown to be slower by 80% in the Cog−PF− compared to that in the Cog+PF+, indicating that FWS was more challenging for Cog−PF−. The FWS test is essential in identifying muscle weakness, decreased functional independence (51), and cognitive decline (52). FWS likely requires a relatively rapid generation of high peak power from the lower extremities’ joints (53) and improvements in lower limbs’ muscle function to enhance joint range of motion, propulsive force, and dynamic stability and thus increase step length (54). Furthermore, modifications in walking speed, such as FWS and SWS, require attention because of the reduced automaticity of gait and increased cortical control due to changes in muscle activity patterns (55, 56). Therefore, SWS and FWS are more challenging gait tasks than PWS (8). It is likely that compared with PWS, these challenging tasks place a greater demand on motor control and, hence, may be more sensitive to age-associated decline (53, 57). Therefore, SWS and FWS may be more challenging gait tasks than PWS for older women with decreasing Cog and PF (8, 58).

For classification between Cog−PF+ and Cog−PF−, walking speed at PWS was selected via stepwise regression to obtain the sensitive cut-off values in the ROC analysis. Cog−PF− significantly decreased walking speed at PWS, compared with Cog+PF+ and Cog−PF+ [Cog+PF+ vs. Cog−PF+: 1.18 ± 0.15 vs. 1.14 ± 0.17 (*p* = 0.467), Cog+PF+ vs. Cog−PF−: 1.18 ± 0.15 vs. 0.97 ± 0.20 (*p* < 0.001), Cog−PF+ vs. Cog−PF−: 1.14 ± 0.17 vs. 0.97 ± 0.20 (*p* < 0.001)]. The optimization of walking observed in the self-preferred speed condition may stem from the interplay between neural and biomechanical mechanisms, aiming to minimize the necessity for active control based on high-level sensory feedback (59). The decline in walking speed among older individuals showed the most robust association with white matter atrophy (6, 60), which has been associated with a decline in executive function among cognitive domains (61). In addition, Yee et al. (13) reported a relationship between decreased walking speed and PF of the lower extremities, assessed using the sit-to-stand test. The decreased strength of the hip flexors and ankle dorsiflexors are thought to influence these changes (52). Therefore, older women with reduced Cog and PF may have difficulty walking even at their preferred speed. We suggest that Cog−PF+ and Cog−PF− may be distinguished using the walking speed at PWS.

### 4.2 Classification by Cog and PF declines using ML approach

Regarding ML approaches, RF demonstrated high accuracy in addressing Cog and PF classification tasks, achieving 85.2% accuracy for Cog+PF+ vs. Cog–PF+ _3 and 91.2 and 91.9% accuracies for Cog+PF+ vs. Cog–PF–_96 and Cog+PF+ vs. Cog–PF–_3, respectively. Meanwhile, QDA had 81.8% accuracy for Cog+PF+ vs. Cog–PF+ _96. Based on these results, the possibility of distinguishing between people with Cog+PF+ and Cog–PF+ and those with Cog+PF+ and Cog–PF–, based on the three speed-based gait characteristics, was somewhat confirmed. RF outperformed all the other classifiers on Cog+PF+ vs. Cog–PF+ and Cog+PF+ vs. Cog–PF– with reduced feature sets. Generally, the output of a model can be influenced by multiple features (62). Overfitting is a common challenge in supervised ML, stemming from constraints such as the limitations of training data—either because of restricted data size or the presence of considerable noise—and the complexity of algorithms, which may require an excessive number of features (63). Therefore, our results indicate that feature selection using stepwise regression eliminates irrelevant features (62).

In contrast, NB and KNN showed accuracies of 71.6 and 72.6%, respectively, for Cog–PF+ vs. Cog–PF– classification tasks. From the results, the possibility of distinguishing between Cog–PF+ and Cog–PF–based on the three speed-based gait characteristics was somewhat confirmed; however, the Cog–PF+ and Cog–PF– classification problem appeared more challenging than the Cog+PF+ and Cog–PF+ as well as Cog+PF+ and Cog–PF– classification problems. No classifier achieved an accuracy above 80%, and the SDs of the accuracies for Cog–PF+ vs. Cog–PF– _96 and Cog–PF+ vs. Cog–PF– _1 were higher for NB, LDA, and QDA compared to the Cog+PF+ vs. Cog+PF– and Cog+PF+ vs. Cog–PF– classifications. We speculate that the observed limitations in classifier performance, with no accuracy exceeding 80%, may be attributed to the study’s relatively small sample size.

The reduced number of samples could potentially lead to misinterpretations in the mathematical optimization process during classifier training, thereby affecting the performances of NB, LDA, and QDA, owing to the inherent characteristics of these classification algorithms. Therefore, to enhance the accuracy of the Cog–PF+ vs. Cog–PF– classification problem, future research should explore the application of advanced deep learning techniques, such as the n-dimensional convolutional neural network and recurrent neural network (62), on time series raw gait data obtained at three different walking speeds. Despite converting raw gait data into the selected 96 features, the risk of losing the crucial information necessary to solve the Cog–PF+ vs. Cog–PF– classification problem remains.

### 4.3 Classification by Cog and MS declines using feature selection through stepwise regression

In the main findings for classification by Cog and MS declines in this study, the selected feature most relevant for the classification of Cog+MS+ and Cog–MS+ was the CV of the left double-support phase at SWS. The ML approach showed that QDA had 77.5% accuracy for Cog+MS+ vs. Cog–MS+ _96 and RF had 64.0% accuracy for Cog+MS+ vs. Cog–MS+ _1. In the classification of Cog+MS+ and Cog–MS–, the two most relevant selected features were the left-step time at PWS and GA at SWS. The ML approach showed that SVM solved the Cog+MS+ and Cog–MS– classification problems with 93.6% accuracy for Cog+MS+ vs. Cog–MS–_96 and Cog+MS+ vs. Cog–MS–_2. In the classification of Cog–MS+ and Cog–MS–, the most relevant selected feature was the CV of the right single-support phase at the PWS. The ML approach showed that QDA had 80.1% accuracy for Cog–MS+ vs. Cog–MS– _96 and LR had 70.8% accuracy for Cog–MS+ vs. Cog–MS– _1. These findings are discussed in detail below.

In our study on the classification of Cog+MS+ and Cog–MS+, the CV of the left double support phase at SWS was selected through stepwise regression to obtain the sensitive cutoff values in the ROC analysis. Stride-to-stride variability, quantified by the coefficient of variation, serves as a metric for gauging the reproducibility of coordinated limb movements from one stride to the next (64). Previous studies have strongly associated gait variability with Cog (15, 17). Our study showed that a higher CV of the left double-support phase was associated with a lower Cog at SWS in older women. These results may be linked to executive dysfunction in the cortical sensorimotor control (17, 65, 66), and this dysfunction could be attributed to the reduced hippocampal volume and impaired function observed in older adults (67). Executive function is associated with the initiation and modulation of gait performance (68). Therefore, the observed high gait variability may be attributed to the stride-to-stride fluctuations when walking, which involve the generation of force using muscles with a partial summation of overlapping twitches (58). This high gait variability may result from executive dysfunction during modulation, which is particularly evident in SWS.

For Cog+MS+ and Cog−MS − classification, left step time at PWS and GA at SWS were selected via stepwise regression to obtain the sensitive cut-off values in the ROC analysis. Previous studies have associated longer step time with Cog decline (47), suggesting that this result may be influenced by the impact of the brainstem and spinal cord mechanisms, which activate and control lower limb muscles and gait movements (69). In addition, previous studies have reported an increase in the mean step time in older adults, indicating a preference for an extended step time as a compensatory strategy for muscle weakness (70, 71) and balance impairments (66, 72, 73). Meanwhile, the GA at SWS was significantly lower for the Cog−MS− group, compared to that for the Cog+MS+ group. The aging process is known to increase the demand for the prefrontal cortex during motor control (74). Individuals in the low Cog and MS groups exhibited lower gait abilities than those in the high-functioning group (19, 44). Specifically, older adults with reduced gait ability demonstrated increased prefrontal cortex activation during challenging tasks such as FWS and SWS (75). These findings suggest that greater prefrontal cortex activation is required during walking tasks that demand cognitive engagement with speed variations, which may be a compensatory mechanism to increase the level of motor task performance (76). In addition, SWS employs a strategy of increasing the medial displacement of the center of mass to enhance the base of support, aiming to maintain dynamic balance (77). Therefore, the results of GA at SWS in the Cog−MS− group compared with those in the Cog+MS+ group may be attributed to these compensatory strategies.

For the classification of the Cog−MS+ and Cog−MS− groups, the CV of the right single support phase at PWS was selected via stepwise regression to obtain the sensitive cut-off values in the ROC analysis. However, the association between muscle strength and gait variability has not been extensively studied. Previous studies have found that lower extremity strength is associated with the CV of step time (46) and CV of step width (78). In addition, Bogen et al. (18) reported an association between lower and upper extremity strength measurements between anterioposterior, mediolateral, and vertical gait variability. While these results cannot be directly compared with those of our study, it appears that, despite gait being an activity that does not require maximal strength, lower- and upper-extremity strength may contribute to gait variability (18). Therefore, the CV of the support phase at PWS may indicate decreased MS in older women with decreasing Cog.

### 4.4 Classification by Cog and MS declines using ML approach

Regarding ML approaches for classifying Cog and MS declines, analyzing all 96 features was found to yield higher accuracy than models based on selected features through stepwise regression. These results show that the 96 features influenced the group classifications more than the selected features. Including many gait features helps to mitigate the bias associated with selecting variables for input in ML analyses. This ML analysis is particularly beneficial when dealing with high dimensionality and non-linear associations among input variables within a relatively small sample size (1). Interestingly, SVM demonstrated high accuracy in addressing Cog+MS+ and Cog–MS– classifications, achieving 93.6% accuracy for Cog+MS+ vs. Cog–MS–_96 and Cog+MS+ vs. Cog–MS–_2. These results indicate the possibility of distinguishing between Cog+MS+ and Cog–MS– based on the three speed-based gait characteristics. Additionally, it was observed that group classification is possible not only with all 96 features but also with selected features, specifically the left-step time for PWS and GA for SWS. Therefore, our results demonstrate that the feature selection process using stepwise regression eliminated irrelevant features in the classification of Cog+MS+ and Cog–MS–.

The findings of the present study have several important implications. First, the ML approach used to resolve this study’s Cog and PF classification problems showed similar results when using gait features selected through gait analysis based on three walking speeds. This result may help understand the gait characteristics and classify older women with Cog and PF declines based on certain main factors of the spatiotemporal and variability features at the three walking speeds. Second, the classification of Cog+PF+ and Cog–PF– showed the highest accuracy of 91.9% in the results of the ML approach using selected gait features. Third, the classification of Cog+MS+ and Cog–MS– groups showed the highest accuracy of 93.6% in the results of the ML approach using selected gait features. This ML analysis of the critical factors selected among the three speed-based gait characteristics can help provide information regarding the Cog, PF, and MS declines conditions of older women.

Our study has several limitations. First, our study used MMSE scores, which are known to have relatively lower sensitivity (79). Nevertheless, the MMSE score is a widely utilized screening tool for dementia, assessing global cognitive function and aiding in clinical evaluations. Further research is required to address these issues. Second, this study included participants who were not diagnosed with dementia and had normal cognitive function. Despite assuming this status, we speculated that some participants might have had a diagnostic level of dementia. Third, we exclude direct comparisons with groups with no signs of Cog decline but PF and MS declines (Cog+PF– and Cog+MS–), as this was beyond the purpose of this study. Although 103 participants in the Cog+PF– and Cog+MS– were also recruited, these groups may be unreliable due to sample size imbalance and data bias problems in the analyses. Lastly, the sample sizes of Cog–PF+, Cog–PF–, Cog–MS+, and Cog–MS– were relatively small compared to those of Cog+PF+ and Cog+MS+. Despite using the random oversampling technique to address the imbalanced dataset, the insufficient sample size may have led to the instability in the classification performance for NB, LDA, and QDA, as mentioned earlier.

## 5 Conclusion

In conclusion, our findings highlight the potential methods for identifying using various speed-based gait characteristics in older women with the declines of PF and MS simultaneously with Cog decline. The ML approach showed that RF had 91.9% accuracy for the Cog+PF+ and Cog–PF– classification problems when using the three selected features (walking speed and CV of left double support phase at FWS and right double support phase at SWS). In addition, SVM showed the highest classification accuracy of 93.6% for the Cog+MS+ and Cog–MS– classification problems using two selected features (left step time at PWS and GA at SWS). Therefore, our results suggest a proof of concept that ML techniques using gait features based on the three walking speeds might represent a reliable surrogate biomarker for Cog, PF, and MS declines. Our findings are useful in assessing diminishing physical function in older adults and could potentially serve as valuable tools in clinical settings to evaluate the efficacy of interventions aimed at preventing or delaying cognitive decline. Future studies need to develop ML techniques that incorporate important factors when evaluating the declines in Cog, PF, and MS and include a larger sample size to enhance the predictive capability for categorizing older women showing declines in Cog, PF, and MS.

## Data availability statement

The datasets presented in this study can be found in online repositories. The names of the repository/repositories and accession number(s) can be found in the article/Supplementary material.

## Ethics statement

The studies involving humans were approved by Institutional review board at Dong-A University (IRB number: 2–104709–AB–N–01–201808–HR–023–02). The studies were conducted in accordance with the local legislation and institutional requirements. The participants provided their written informed consent to participate in this study.

## Author contributions

BK: Conceptualization, Data curation, Formal analysis, Investigation, Methodology, Writing – original draft, Writing – review & editing. CY: Conceptualization, Data curation, Formal analysis, Funding acquisition, Methodology, Supervision, Writing – original draft, Writing – review & editing. HP: Conceptualization, Data curation, Formal analysis, Investigation, Methodology, Writing – original draft, Writing – review & editing. HC: Conceptualization, Data curation, Formal analysis, Methodology, Writing – original draft, Writing – review & editing. SS: Conceptualization, Data curation, Formal analysis, Investigation, Methodology, Writing – original draft, Writing – review & editing.

## Glossary

Cog: Cognitive function
PF: Physical function
MS: Muscle strength
ML: Machine-learning
Cog+PF+: Group without signs of declines in Cog and PF
Cog−PF+: Group with Cog decline but no signs of PF declines
Cog−PF−: Group with signs of declines in Cog and PF
Cog+MS+: Group without signs of declines in Cog and MS
Cog−MS+: Group with Cog decline but no signs of MS declines
Cog−MS−: Group with signs of declines in Cog and MS
IRB: Institutional review board
MMSE: Mini-mental status examination
FSTS: Five times sit-to-stand
HGS: Handgrip strength
PWS: Preferred walking speed
SWS: Slower walking speed
FWS: Faster walking speed
IMU: Inertial measurement unit
GV: Gait variability
CV: Coefficient of variance
GA: Gait asymmetry
PCI: Phase coordination index
ANOVA: Analysis of variance
SD: Standard deviation
ICC: Intra-class correlation coefficient
LoA: Limits of agreement
BMI: Body mass index
OR: Odds ratios
ROC: Receiver operating characteristic
AUC: Areas under the curve
LR: Logistic regression
KNN: K-nearest neighbors
NB: Naїve Bayes
LDA: Linear discriminant analysis
QDA: Quadratic discriminant analysis
SVM: Support vector machine
RF: Random forest
Cog+PF+ vs. Cog−PF + _96: Cog+PF+ vs. Cog−PF+ with 96 features
Cog+PF+ vs. Cog−PF + _3: Cog+PF+ vs. Cog−PF+ with 3 features selected through stepwise regression, Cog+PF+ vs. Cog−PF − _96
Cog+PF+ vs. Cog−PF− with 96 features, Cog+PF+ vs. Cog−PF − _3: Cog+PF+ vs. Cog−PF− with 3 features selected through stepwise regression
Cog−PF+ vs. Cog−PF − _96: Cog−PF+ vs. Cog−PF− with 96 features
Cog−PF+ vs. Cog−PF − _1: Cog−PF+ vs. Cog−PF− with 1 feature selected through stepwise regression
Cog+MS+ vs. Cog−MS + _96: Cog+MS+ vs. Cog−MS+ with 96 features
Cog+MS+ vs. Cog−MS + _1: Cog+MS+ vs. Cog−MS+ with 1 feature selected through stepwise regression, Cog+MS+ vs. Cog−MS − _96
Cog+MS+ vs. Cog−MS− with 96 features, Cog+MS+ vs. Cog−MS − _2: Cog+MS+ vs. Cog−MS− with 2 features selected through stepwise regression
Cog−MS+ vs. Cog−MS − _96: Cog−MS+ vs. Cog−MS− with 96 features
Cog−MS+ vs. Cog−MS − _1: Cog−MS+ vs. Cog−MS− with 1 feature selected through stepwise regression.
