# Supplementary material for: Machine learning approach to classifying declines of physical function and muscle strength associated with cognitive function in older women: gait characteristics based on three speeds
Source: Front Public Health. 2024 Jun 12;12:1376736. doi: 10.3389/fpubh.2024.1376736 (PMC11232496; doi:10.3389/fpubh.2024.1376736)
Supplement: Supplementary file 1 [file Data_Sheet_1.docx]

| **Supplementary table 1**. Precision, recall, and F1 score of the 7 classifiers for 6 cases by Cog and PF classification problems | | | | | | | |
| --- | --- | --- | --- | --- | --- | --- | --- |
|  | **ML techniques** | **Cog+PF+ vs. Cog–PF+**  **(w/96 Features)** | **Cog+PF+ vs. Cog–PF+**  **(w/3 Features)** | **Cog+PF+ vs. Cog–PF–**  **(w/96 Features)** | **Cog+PF+ vs. Cog–PF–**  **(w/3 Features)** | **Cog–PF+ vs. Cog–PF–**  **(w/96 Features)** | **Cog–PF+ vs. Cog–PF–**  **(w/1 Feature)** |
| Precision | LR | 72.8 ± 6.7 | 69.0 ± 11.3 | 85.8 ± 5.4 | 87.5 ± 2.7 | 68.9 ± 6.6 | 53.3 ± 28.6 |
|  | KNN | 74.1 ± 10.6 | 66.8 ± 4.4 | 81.0 ± 7.3 | 88.7 ± 5.1 | 72.2 ± 9.0 | 76.8 ± 8.2 |
|  | NB | 69.7 ± 8.2 | 69.8 ± 10.8 | 84.4 ± 5.6 | 85.3 ± 2.8 | 74.9 ± 11.3 | 67.8 ± 13.7 |
|  | LDA | 66.3 ± 6.0 | 67.6 ± 15.3 | 81.3 ± 4.8 | 87.6 ± 4.1 | 54.4 ± 23.7 | 63.7 ± 14.5 |
|  | QDA | 86.1 ± 1.7 | 71.2 ± 9.8 | 91.8 ± 5.3 | 85.9 ± 3.4 | 44.8 ± 10.8 | 67.8 ± 13.7 |
|  | SVM | 81.6 ± 5.3 | 81.6 ± 5.3 | 91.3 ± 5.3 | 88.1 ± 5.7 | 70.7 ± 5.5 | 75.3 ± 6.5 |
|  | RF | 80.5 ± 5.0 | 87.0 ± 5.6 | 91.4 ± 5.1 | 92.1 ± 1.8 | 69.8 ± 6.4 | 67.0 ± 3.7 |
| Recall | LR | 71.7 ± 6.7 | 64.8 ± 11.2 | 85.1 ± 5.9 | 87.2 ± 2.7 | 67.9 ± 7.5 | 53.8 ± 8.9 |
|  | KNN | 71.0 ± 9.5 | 64.9 ± 2.1 | 80.5 ± 7.4 | 88.6 ± 5.1 | 67.9 ± 8.8 | 72.6 ± 6.5 |
|  | NB | 68.8 ± 7.9 | 67.6 ± 10.1 | 83.0 ± 5.6 | 84.4 ± 2.0 | 71.6 ± 10.2 | 66.6 ± 12.3 |
|  | LDA | 64.9 ± 4.8 | 65.5 ± 14.7 | 80.4 ± 8.1 | 87.2 ± 4.4 | 53.7 ± 21.5 | 63.2 ± 13.8 |
|  | QDA | 81.8 ± 2.9 | 68.3 ± 9.0 | 91.2 ± 5.3 | 84.4 ± 4.0 | 45.4 ± 9.8 | 66.6 ± 12.3 |
|  | SVM | 69.0 ± 13.0 | 69.0 ± 13.0 | 91.2 ± 5.3 | 87.9 ± 5.7 | 69.1 ± 6.1 | 69.1 ± 4.4 |
|  | RF | 79.7 ± 4.7 | 85.2 ± 6.4 | 91.2 ± 5.2 | 91.9 ± 1.7 | 69.1 ± 7.4 | 65.4 ± 5.1 |
| F1 scroe | LR | 71.3 ± 6.8 | 62.0 ± 14.1 | 85.0 ± 5.9 | 87.1 ± 2.8 | 67.7 ± 7.8 | 42.6 ± 15.7 |
|  | KNN | 70.0 ± 9.8 | 64.1 ± 1.5 | 80.4 ± 7.5 | 88.5 ± 5.1 | 65.9 ± 10.6 | 71.5 ± 7.0 |
|  | NB | 68.5 ± 8.0 | 66.6 ± 10.7 | 82.9 ± 5.8 | 84.3 ± 5.0 | 70.7 ± 10.4 | 66.5 ± 12.3 |
|  | LDA | 64.1 ± 4.9 | 64.5 ± 15.1 | 80.3 ± 8.2 | 87.1 ± 4.5 | 53.1 ± 22.1 | 62.6 ± 14.8 |
|  | QDA | 81.2 ± 3.3 | 67.0 ± 10.2 | 91.1 ± 5.3 | 84.2 ± 4.2 | 42.3 ± 10.0 | 66.5 ± 12.3 |
|  | SVM | 64.1 ± 17.2 | 64.1 ± 17.2 | 91.2 ± 5.2 | 87.8 ± 5.7 | 68.5 ± 6.4 | 67.4 ± 5.3 |
|  | RF | 79.7 ± 4.7 | 85.0 ± 6.6 | 91.2 ± 5.2 | 91.9 ± 1.7 | 68.9 ± 7.7 | 64.7 ± 6.2 |
| Precision, recall, and F1 score are represented as mean (%) ± standard deviation (%). *ML,* machine learning; *Cog,* cognitive function; *PF*, physical function; *LR*, logistic regression; *KNN*, k­nearest neighbors; *NB*, naїve bayes; *LDA*, linear discriminant analysis; *QDA*, quadratic discriminant analysis; *SVM,* support vector machine; *RF*, random forest | | | | | | | |

| **Supplementary table 2**. Precision, recall, and F1 score of the 7 classifiers for 6 cases by Cog and MS classification problems | | | | | | | |
| --- | --- | --- | --- | --- | --- | --- | --- |
|  | **ML techniques** | **Cog+MS+ vs. Cog–MS+**  **(w/96 Features)** | **Cog+MS+ vs. Cog–MS+**  **(w/1 Feature)** | **Cog+MS+ vs. Cog–MS–**  **(w/96 Features)** | **Cog+MS+ vs. Cog–MS–**  **(w/2 Features)** | **Cog–MS+ vs. Cog–MS–**  **(w/96 Features)** | **Cog–MS+ vs. Cog–MS–**  **(w/1 Feature)** |
| Precision | LR | 66.5 ± 9.5 | 63.3 ± 11.8 | 84.1 ± 6.5 | 56.0 ± 6.3 | 64.3 ± 15.8 | 71.8 ± 9.7 |
|  | KNN | 70.7 ± 5.9 | 59.5 ± 10.9 | 78.0 ± 3.3 | 66.2 ± 5.6 | 65.5 ± 10.3 | 69.7 ± 6.9 |
|  | NB | 63.8 ± 7.9 | 57.0 ± 15.4 | 72.9 ± 6.7 | 60.2 ± 11.3 | 69.4 ± 16.8 | 69.7 ± 8.6 |
|  | LDA | 65.8 ± 9.8 | 62.0 ± 12.3 | 83.3 ± 6.9 | 58.7 ± 8.9 | 76.0 ± 10.2 | 67.7 ± 11.1 |
|  | QDA | 82.9 ± 4.2 | 59.9 ± 12.0 | 94.6 ± 4.1 | 59.7 ± 5.7 | 84.1 ± 5.4 | 70.4 ± 7.8 |
|  | SVM | 36.7 ± 29.8 | 35.9 ± 27.8 | 94.6 ± 4.1 | 94.6 ± 4.1 | 36.3 ± 31.0 | 70.7 ± 8.4 |
|  | RF | 70.2 ± 6.8 | 64.3 ± 10.3 | 86.1 ± 5.7 | 80.5 ± 4.7 | 74.0 ± 10.0 | 67.2 ± 6.2 |
| Recall | LR | 65.4 ± 9.2 | 62.2 ± 11.6 | 82.0 ± 6.3 | 55.2 ± 5.4 | 62.5 ± 15.1 | 70.8 ± 9.5 |
|  | KNN | 69.9 ± 6.2 | 58.3 ± 9.9 | 77.6 ± 3.6 | 66.0 ± 5.8 | 63.6 ± 9.3 | 68.7 ± 6.7 |
|  | NB | 62.8 ± 8.0 | 55.8 ± 10.4 | 71.7 ± 6.0 | 55.8 ± 5.1 | 64.6 ± 13.6 | 60.4 ± 2.6 |
|  | LDA | 64.7 ± 8.4 | 61.0 ± 12.2 | 80.8 ± 6.0 | 58.4 ± 8.5 | 72.9 ± 9.5 | 63.6 ± 8.0 |
|  | QDA | 77.5 ± 7.7 | 58.4 ± 11.2 | 93.6 ± 5.1 | 57.7 ± 5.2 | 80.1 ± 7.0 | 60.5 ± 3.8 |
|  | SVM | 56.2 ± 17.5 | 55.6 ± 16.1 | 93.6 ± 5.1 | 93.6 ± 5.1 | 55.9 ± 19.1 | 68.8 ± 8.1 |
|  | RF | 68.5 ± 6.0 | 64.0 ± 10.0 | 84.0 ± 6.7 | 77.6 ± 6.0 | 71.9 ± 10.6 | 66.6 ± 5.2 |
| F1 scroe | LR | 64.7 ± 9.3 | 60.8 ± 12.6 | 81.7 ± 6.7 | 54.7 ± 4.9 | 61.8 ± 15.1 | 70.5 ± 9.4 |
|  | KNN | 69.5 ± 6.4 | 56.9 ± 10.4 | 77.5 ± 3.7 | 66.0 ± 5.9 | 62.8 ± 9.1 | 68.3 ± 6.7 |
|  | NB | 61.7 ± 8.7 | 51.0 ± 12.9 | 71.4 ± 6.1 | 53.2 ± 5.1 | 63.1 ± 13.4 | 56.2 ± 3.8 |
|  | LDA | 64.4 ± 8.1 | 59.2 ± 13.3 | 80.4 ± 6.1 | 58.3 ± 8.5 | 72.1 ± 9.5 | 61.9 ± 7.8 |
|  | QDA | 76.3 ± 8.7 | 54.9 ± 13.1 | 93.5 ± 5.2 | 55.3 ± 6.5 | 79.3 ± 7.8 | 55.2 ± 5.5 |
|  | SVM | 42.7 ± 24.9 | 42.1 ± 23.6 | 93.5 ± 5.2 | 93.5 ± 5.2 | 42.3 ± 26.6 | 68.0 ± 8.1 |
|  | RF | 68.0 ± 6.0 | 63.6 ± 10.5 | 83.7 ± 7.0 | 76.9 ± 6.7 | 71.2 ± 11.1 | 66.3 ± 5.0 |
| Precision, recall, and F1 score are represented as mean (%) ± standard deviation (%). *ML,* machine learning; *Cog,* cognitive function; *MS*, muscle strength; *LR*, logistic regression; *KNN*, k­nearest neighbors; *NB*, naїve bayes; *LDA*, linear discriminant analysis; *QDA*, quadratic discriminant analysis; *SVM,* support vector machine; *RF*, random forest | | | | | | | |
